# Public Vaccination Reluctance: What Makes Us Change Our Minds? Results of A Longitudinal Cohort Survey

**DOI:** 10.3390/vaccines10071081

**Published:** 2022-07-05

**Authors:** Paweł Waszkiewicz, Piotr Lewulis, Michał Górski, Adam Czarnecki, Wojciech Feleszko

**Affiliations:** 1 Department of Criminalistics, Faculty of Law and Administration, University of Warsaw, Krakowskie Przedmieście 26/28, 00-927 Warsaw, Poland; p.lewulis@wpia.uw.edu.pl (P.L.); mgorski@student.uw.edu.pl (M.G.); 2 ARC Rynek i Opinia, Market Research Institute, Słowackiego 12, 01-627 Warsaw, Poland; adam.czarnecki@arc.com.pl; 3 Department of Pediatric Pulmonology and Allergy, Medical University of Warsaw, Żwirki i Wigury 63A, 02-091 Warsaw, Poland; wojciech.feleszko@wum.edu.pl

**Keywords:** COVID-19, vaccines, vaccine hesitancy

## Abstract

The paper presents a longitudinal cohort survey on the public acceptability of COVID-19 vaccination and real vaccination levels. A survey on a representative sample of adult Polish citizens (*n* = 1066) was conducted in June 2020 and, one year later, the same group was approached to compare the hypothetical declarations with the real vaccination decisions (*n* = 438). A significant part of the group that declared reluctance and hesitation toward COVID-19 vaccination before that vaccination was available actually got vaccinated or plans to get vaccinated. Those respondents were asked about the reasons for changing their attitudes. Among the previously vaccine-reluctant individuals, the main reasons included concern about their health and safety (50%) and their desire to travel (26.6%). Vaccine-hesitant individuals also indicated health and safety as their primary concern (69%), as well as the pursuit of herd immunity and a notion of common social safety (12.6%). The main factors helping to increase vaccination acceptance are based on a self-centered pursuit of safety and freedom from restrictions. The survey results may help to prepare a more effective vaccination campaign.

## 1. Introduction

December 2021 marked two significant anniversaries associated with the COVID-19 global pandemic: the first case of COVID-19 was identified in December 2019 and, in December 2020, several countries approved the deployment of vaccines against the virus. The development and production of vaccines at an unprecedented pace made rapid immunization possible for billions of people worldwide. New antiviral drugs could become a game-changer in the coming months [1,2], but massive vaccination remains one of the most important preventive measures against the SARS-CoV-2 virus. With the Omicron variant and new BA.2, BA.4 and 5 subvariants spreading much faster than previous versions of the coronavirus, it is unlikely that vaccines will help countries to achieve so-called herd immunity against COVID-19, and the mathematical models on which these estimates are based make numerous assumptions that may not be met in the real world. Nevertheless, the COVID-19 vaccination still offers protection against symptomatic and fatal SARS-CoV-2 infection, even as the Omicron variant became predominant [3]. However, COVID-19 immunization rates are still unsatisfactory even in the G20’s most developed countries. By February 2022, 64% of eligible Americans and 71% of eligible EU citizens [4] were fully vaccinated. Notably, the rates of vaccination are affected by country-specific variables, many of economic or organizational nature. Existing challenges for successful vaccination programs are associated with the difficulties in the mass manufacturing of vaccines and their fair distribution [5]. Such issues are, of course, of great importance and each country may be subject to a separate in-depth analysis in this context. However, vaccine hesitancy is still a major factor slowing down the efforts to combat the pandemic in many regions [6,7]. Various anti-vaccination stances are often referred to as falling under the umbrella term of ‘vaccine hesitancy’, and of course low vaccine hesitancy levels do not mean high vaccine demand, as it may mean strong vaccine reluctance [8]. Within the scope of this work, both vaccine hesitancy and reluctance are treated jointly as attitudes disrupting COVID-19 vaccination programs. In June 2020, our overview of international attitudes towards COVID-19 vaccination indicated that low vaccination acceptance is common in many nations [9]. At the time, the question of vaccination willingness was hypothetical; no COVID-19 vaccine had been fully developed and approved for use. A year after vaccines have been deployed, hesitancy and reluctance continue to result in hundreds of millions of people worldwide deciding not to vaccinate against COVID-19. To effectively promote vaccination, it would be beneficial to accurately identify the reasons behind such individual changes. It is established that, in combating vaccine-hesitancy and reluctance, there is no ‘one size fits all’ method, and it is crucial to understand the determinants and correlations associated with attitudes towards vaccination in groups of hesitant individuals [10]. Social campaigns and guidelines aiming to encourage vaccination among those that are hesitant or reluctant to do so are being designed by various public health professionals and divisions (e.g., the CDC [11], GAVI [12], and RAND [13]). However, due to the understandable scarcity of existing research specific to COVID-19 vaccination hesitancy [13,14,15], these policies are based on empirically unverified reasons for changing individual attitudes towards COVID-19 vaccination. Most of the pre-COVID-19 research on the individual reasons behind vaccine hesitancy was focused on parental attitudes towards immunizing children [16]. Now, the factors shaping the attitudes and decision-making processes may be different. For example, the novelty of the disease itself seems to have an additional negative psychological effect [17,18]. Among common reasons given by people who say they are unlikely to vaccinate against COVID-19 are concerns about its unknown future side effects [19], but such studies to date have been based mostly on declarations of intentions. Even extensive reviews of studies that focus on vaccination hesitancy among certain groups conclude the need for more research [20]. To address this issue, we conducted a longitudinal survey study to determine the extent of individual declarations of intent and subsequent real-life actions. The main goal of this article is, therefore, based on this study, to identify what are the reasons behind changes in individual decisions to vaccinate against COVID-19.

## 2. Methodology

We conducted the survey study in Poland using an online opt-in panel operated by the ARC Rynek i Opinia Independent Research Institute. The respondents were selected from a national online panel—epanel.pl, a well-established Polish database with 75,000 available respondents. Upon the registration procedure, all the panel members were informed about the purpose of the panel membership and the anonymity of their participation. Respondents were specifically informed about the purpose of each study before they decide whether they want to participate in it by filling in the questionnaire. Respondents receive small monetary compensation for participation in each project completed on epanel.pl. The research procedure meets the standards of ESOMAR (the World Association for Social, Opinion and Market Research) and GRBN (the Global Research Business Network) guidelines for online sample quality [21].

The survey had two phases: the first on 2–9 June 2020 and the second on 4–10 August 2021. For both phases, Cadas software was used for online self-completed questionnaires (CADAS Software Sp. z o.o., Warsaw, Poland). The software controls the flow of the responses and the respondent cannot skip to the next question without answering the previous one. The pilot study on a sample of *n* = 50 respondents was used to test the questionnaire. The answers of the respondents were verified and slight modifications were applied. The data file provided by the Research Institute contained no personally identifying information. The survey was reviewed and approved by the Bioethics Committee at the Medical University of Warsaw.

The first phase, on 2–9 June 2020, included a sample of *n* = 1066 consenting adults. Quota non-probability sampling and statistical weighting by gender, age, region, city size, and education levels were applied to make the sample representative of Poland’s entire adult population (aged 18–65, as it is described by the available statistical data (GUS, Statistics Poland [22]). The smallest cell in quotas per age and sex were no less than 50 respondents. There is no simple formula for determining the appropriate non-probability sample size for online studies [23].

The questionnaire targeted the respondents’ declared will to be vaccinated against COVID-19 once a vaccine is available to the public, which was hypothetical at the time. A selection of answers was provided: Yes (‘I am willing to vaccinate’), No (‘I will not get vaccinated’), and I do not know (‘I am not sure’).

In the second phase of the survey study, on 4–10 August 2021, a follow-up survey was conducted using the same tools and methodology. At this time, the survey participants were asked whether or not they were vaccinated against COVID-19 (and if not, whether they were planning to be within the next six months). We invited the same individuals to participate in the study. Out of the original sample, *n* = 438 (41%) agreed to participate in the follow-up survey. To maximize the response rate, several invitations were sent to participants of the first phase over two weeks. To enable reliable findings, only those who took part in both phases of the survey were included in the analysis. Importantly, the participants were assigned individual (yet anonymized) codes allowing us to link responses across the study phases. A quantitative analysis of these answers provided a change-in-attitudes scale toward COVID-19 vaccination. In both phases, respondents were informed about the anonymization of responses.

In the follow-up survey, all the participants who declared that they got vaccinated or were planning to do so were asked about their motivation (‘Why did you get vaccinated against COVID-19’ or ‘Why are you planning to get vaccinated against COVID-19’). The question was open-ended (a freeform text box was provided) and non-mandatory. A total of *n* = 301 individuals (out of 307 eligible respondents) provided their responses, which were subject to the analysis allowing us to determine the categories of individual motivations leading to COVID-19 vaccination.

For context, we also compared the quantitative data on COVID-19 vaccination willingness expressed in 2020 in various countries based on the previously published data [9], with the current vaccination levels in the same countries based on the data available in February 2022 [4] to determine whether the vaccination coverage corresponds with previous declarations on a general level.

The results were checked using the chi-squared test. This was conducted in two phases: Firstly, an overall test was conducted to check if there is an overall difference in the distribution of reasons for vaccination. This test was statistically significant, with χ = 35.03 and *p* < 0.001. In the second step, pairwise tests were performed to check which groups had different motives for vaccination. For multiple testing, the Holm adjustment method was used. Statistical analysis was performed in R version 4.1.2, and graphs were created using ggplot2 package [24].

## 3. Results

**Figure 1:**
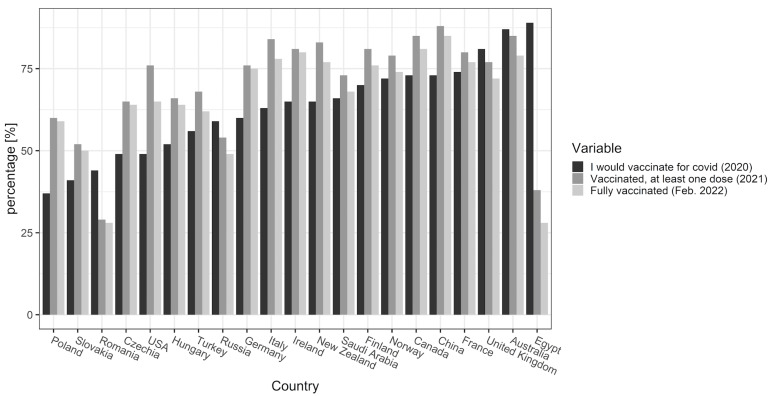
An international overview of the willingness to vaccinate against COVID-10 (in 2020) and the actual vaccination rate in 2021 and 2022.

The comparison of quantitative data on vaccination willingness (2020) with the current (at the time of writing—February 2022) vaccination rates show consistency between these rates in some countries, but divergence in others (Figure 1). COVID-19 vaccination is not mandatory (although it is planned in some countries at the time of writing), so the discrepancies between the willingness to vaccinate and current actual immunization rates indicate changes in individual attitudes towards the issue. Further analysis was devoted to determining the possible reasons for such changes in the Polish population.

In 2020, 169 (38%) individuals were willing to vaccinate and 111 (25%) were vaccine-hesitant. The remaining 158 (36%) were reluctant. By August 2021, 233 (53%) were fully vaccinated. A total of 15 people received one dose and were willing to get a second one, and 5 did not want to be fully vaccinated despite already obtaining the first dose (they are 3.42% and 1.14%, respectively of the overall sample). A total of 54 subjects did not vaccinate, but were willing to (12.33%), and 131 (29.91%) did not vaccinate at all and were not planning to do so.

As shown in Table 1, in the 2020 survey, a total of 38% of respondents declared their willingness to vaccinate against COVID-19 (Group A). Individuals in this group usually (79%) followed through with receiving their immunization by August 2021 or at least were planning to do so within the next 6 months (12%). Out of the 25% of those who declared COVID-19 vaccination reluctance in 2020 (Group B), the majority (67%) remained unwilling and unplanning to vaccinate, but 27% did change their attitude and got vaccinated by August 2021, and another 6% declared that they are planning to do so. Notably, the majority of individuals who were not sure about their willingness to vaccinate in 2020 (Group C, comprising 36% of respondents) had received at least one dose of a COVID-19 vaccine (53%) or planned to be vaccinated (16%) in 2021. These findings demonstrate that individual decisions on vaccination may change over time, potentially leading to a rise in vaccination acceptance.

In the follow-up survey, vaccinated individuals (and those who were at least planning to get vaccinated against COVID-19) were asked to disclose the reasons behind their decision to vaccinate in an open-ended question (Supplementary File). Based on an analysis of responses, the emerging categories included concerns for own health and safety, the desire to travel more easily and to end the restrictions set in place against COVID-19, peer pressure and external persuasion, the pursuit of herd immunity, and the notion of common safety, as well as other reasons. Thanks to the survey methodology, it was possible to link these individual responses with attitudes towards COVID-19 vaccination expressed by the same respondents in the first phase of the survey. This was conducted to understand the reasons motivating individuals’ choices to vaccinate despite previous hesitation or reluctance.

As shown in Table 2, among the previously vaccine-reluctant individuals (Group B), the main reasons behind their willingness to vaccinate in 2021 included concerns about their health and safety (46%) and their desire to travel (27%)—which is significantly more difficult without COVID-19 vaccination certification. Vaccine-hesitant individuals (Group C) also indicated health and safety as their primary concern (63%). Interestingly, among vaccine-acceptant individuals (Group A), concerns for their own health and safety were also the most frequently reported reason for COVID-19 vaccination (70%), but the desire to end the restrictions and facilitate travel played a significantly smaller role within this group (7%).

The results show that only those that were strictly against vaccination in 2020 were different in their motivation to finally consider vaccines. There was no statistically significant difference between those willing and vaccine-hesitant in 2020 (χ =5.59, adjusted *p*-value = 0.348). Those that were against vaccination in 2020, but later did get vaccinated or were considering it, were statistically different in their motivation from the other two groups (χ = 28, *p* < 0.001, χ = 15.6, *p* < 0.05 for comparison with those willing to vaccinate and vaccine hesitant, respectively).

To check which specific motivations are related to which attitude towards vaccination in 2020, both variables were dummy-coded and then the Spearman correlation was calculated. The results are shown in Table 3.

## 4. Conclusions

A comparative analysis of the results of both stages of the survey confirms that people, over time, really do change their decisions about vaccinating against COVID-19. This is not particularly surprising in itself. However, the quantitative results of the survey supplement the existing knowledge on the subject as they indicate the exact scale of such changes among specific individuals participating in the study approximately one year apart.

To date, the specific reasons for a change in the attitude of those who were initially reluctant or hesitant to COVID-19 vaccination have been under-researched. Even methodologically sound longitudinal studies focus on the attitudes toward vaccination, not the real or declared vaccination rate changes [25,26,27]. Among variables that may play a significant role in attitudes towards vaccination, the role of the healthcare providers is promising. They serve both as the important source of information and the role models for their patients [28,29,30].

The key findings from this study relate to the reasons why specific individuals chose to get vaccinated against COVID-19 despite prior reluctance or hesitation. The results of the survey suggest that the main factors helping to increase vaccination in all subgroups (vaccine acceptant, reluctant, and hesitant) are based on a self-centered pursuit of one’s own health and safety. The second most important source of motivation, especially relevant for vaccine-reluctant individuals, is the desire to gain freedom from restrictions put in force against the spread of COVID-19 (especially if they hinder one’s ability to travel internationally). The correlation analysis showed that those two motives were also the only ones where the two groups displayed a significant difference. Participants willing to vaccinate were more influenced by concerns about their own safety, while avoiding restrictions was more related to the group previously vaccine-reluctant. In our study, participants did not confirm celebrities’ and public figures’ influential power in promoting COVID-19 vaccination. Therefore, immunization promotion strategies should focus more on the personal health risks associated with COVID-19 and the social benefits of vaccination.

Of course, our study has some known methodological limitations that need to be taken into account. The respondents were asked about their attitudes and their real-life decisions; however, due to the anonymity of the CAWI method, the responses cannot be verified. Secondly, the initial sample (in the first phase of the study) was representative of the adult population in Poland (18–65) in 2020. Due to a naturally limited response rate, less than half (41%) of that initial group participated in the second phase of the study. Moreover, online survey methods generally tend to limit the participation of the elderly (>65). Given the importance of vaccination of senior citizens, a lack of insight into their attitudes and decisions surrounding COVID-19 vaccination should be addressed in future surveys. Still, CAWI method with its anonymity and ease of use has its benefits: in contrast to other survey methods (CAPI and CATI), the social desirability bias is greatly limited (respondents may feel less obliged to report behavior seen as favorable by the interviewer). In the case of COVID-19 vaccination, this could easily lead to over-reporting of vaccination willingness (and under-reporting of vaccination hesitancy). As for the recall bias, a longitudinal survey benefits from it: individual respondents may not remember their own responses from the first phase of the study, so it is more difficult for them to manipulate their answers after one year.

Vaccination hesitancy is not a new phenomenon and COVID-19 is not the last global public health threat. Thus, it is essential to understand the factors that influence people’s decisions about vaccination and changes in viewpoints to prepare effective vaccination strategies for a healthy future.

## Figures and Tables

**Table 1:** Intention to vaccinate (2020) versus actual vaccination acceptance (2021).

Responses:	In 2020 (*n* = 438):	In 2021:
**I would vaccinate (Group A)**	38%	(*n* = 169)
I am vaccinated (at least one dose)		79%
I am planning to get vaccinated		12%
I do not plan to vaccinate		9%
**I would not vaccinate (Group B)**	25%	(*n* = 111)
I am vaccinated (at least one dose)		27%
I am planning to get vaccinated		6%
I do not plan to vaccinate		67%
**I do not know/I am not sure (Group C)**	36%	(*n* = 158)
I am vaccinated (at least one dose)		57%
I am planning to get vaccinated		16%
I do not plan to vaccinate		27%

**Table 2:** Reasons for individual change of attitude towards COVID-19 vaccination among those who declared COVID-19 vaccination hesitance or reluctance (in 2020), although vaccinated in 2021.

**Group A: Individuals Willing to Vaccinate in 2020, Who Are Vaccinated or Are Planning to in 2021 (*n* = 151)**	**Given Reasons for COVID-19 Vaccination:**	
	Concerns for own health and safety	70%
	Wanting to travel and to end the restrictions	7%
	Peer pressure and persuasion	3%
	The pursuit of herd immunity and common safety	10%
	I don’t know	1%
	Other reasons	9%
**Group B: Individuals vaccine reluctant in 2020, but are vaccinated or are planning to in 2021 (*n* = 37)**	**Given reasons for COVID-19 vaccination:**	
	Concerns for own health and safety	46%
	Wanting to travel and to end the restrictions	27%
	Peer pressure and persuasion	5%
	The pursuit of herd immunity and common safety	3%
	I don’t know	11%
	Other reasons	8%
**Group C: Individuals vaccine hesitant in 2020, but are vaccinated or are planning to in 2021 (*n* = 113)**	**Given reasons for COVID-19 vaccination:**	
	Concerns for own health and safety	63%
	Wanting to travel and to end the restrictions	12%
	Peer pressure and persuasion	7%
	The pursuit of herd immunity and common safety	11%
	I don’t know	1%
	Other reasons	6%

**Table 3:** Correlation between willingness to vaccinate in 2020 and motivations for vaccination in 2021.

Motivation for Vaccination in 2021	Willingness to Vaccinate in 2020		
	Yes	No	Don’t Know/Hard to Tell
Concerns for own health and safety	0.12 *	−0.14 *	−0.03
Wanting to travel and to end the restrictions	−0.15 *	0.19 **	0.03
The pursuit of herd immunity and common safety	0.02	−0.09	0.04
Peer pressure and persuasion	−0.08	0.01	0.07
Other reasons	0.05	0	−0.05
I don’t know	−0.1	0.24	−0.06

* *p*-value < 0.05; ** *p*-value < 0.01.

## Data Availability

Part of the data available in a publicly accessible repositories (see list of references). The survey data is contained within the article and in the Supplementary Materials.

## Supplementary Materials

The following are available online at https://www.mdpi.com/article/10.3390/vaccines10071081/s1, Supplement File: original questions (in Polish) and translated into English.

## References

[1] Cully M. (2021). A Tale of Two Antiviral Targets-and the COVID-19 Drugs That Bind Them. Nat. Rev. Drug Discov..

[2] Graham F. (2021). Daily Briefing: Pfizer’s COVID Pill Looks Promising. Nature.

[3] Johnson A.G. (2022). COVID-19 Incidence and Death Rates Among Unvaccinated and Fully Vaccinated Adults with and without Booster Doses During Periods of Delta and Omicron Variant Emergence—25 U.S. Jurisdictions, April 4–December 25, 2021. MMWR Morb. Mortal Wkly. Rep..

[4] Ritchie H., Mathieu E., Rodés-Guirao L., Appel C., Giattino C., Ortiz-Ospina E., Hasell J., Macdonald B., Beltekian D., Roser M. Coronavirus Pandemic (COVID-19). Our World Data. https://ourworldindata.org/coronavirus.

[5] Choudhary O.P., Choudhary P., Singh I. (2021). India’s COVID-19 Vaccination Drive: Key Challenges and Resolutions. Lancet Infect. Dis..

[6] Sallam M. (2021). COVID-19 Vaccine Hesitancy Worldwide: A Concise Systematic Review of Vaccine Acceptance Rates. Vaccines.

[7] Dror A.A., Eisenbach N., Taiber S., Morozov N.G., Mizrachi M., Zigron A., Srouji S., Sela E. (2020). Vaccine Hesitancy: The next Challenge in the Fight against COVID-19. Eur. J. Epidemiol..

[8] MacDonald N.E. (2015). Vaccine Hesitancy: Definition, Scope and Determinants. Vaccine.

[9] Feleszko W., Lewulis P., Czarnecki A., Waszkiewicz P. (2021). Flattening the Curve of COVID-19 Vaccine Rejection—An International Overview. Vaccines.

[10] Dubé E., Gagnon D., MacDonald N.E. (2015). Strategies Intended to Address Vaccine Hesitancy: Review of Published Reviews. Vaccine.

[11] CDC 12 COVID-19 Vaccination Strategies for Your Community. https://www.cdc.gov/vaccines/covid-19/vaccinate-with-confidence/community.html.

[12] Mouser A. What Are the Most Effective Ways to Improve Vaccination Rates?. https://www.gavi.org/vaccineswork/what-are-most-effective-ways-improve-vaccination-rates.

[13] Schmitzberger F.F., Scott K.W., Nham W., Mathews K., Schulson L., Fouche S., Berri N., Shehab A., Gupta A., Salhi R.A. (2021). Identifying Strategies to Boost COVID-19 Vaccine Acceptance in the United States.

[14] Horiuchi S., Sakamoto H., Abe S.K., Shinohara R., Kushima M., Otawa S., Yui H., Akiyama Y., Ooka T., Kojima R. (2021). Factors of Parental COVID-19 Vaccine Hesitancy: A Cross Sectional Study in Japan. PLoS ONE.

[15] Machingaidze S., Wiysonge C.S. (2021). Understanding COVID-19 Vaccine Hesitancy. Nat. Med..

[16] Dubé E., Laberge C., Guay M., Bramadat P., Roy R., Bettinger J.A. (2013). Vaccine Hesitancy. Hum. Vaccines Immunother..

[17] Troiano G., Nardi A. (2021). Vaccine Hesitancy in the Era of COVID-19. Public Health.

[18] Chou W.-Y.S., Budenz A. (2020). Considering Emotion in COVID-19 Vaccine Communication: Addressing Vaccine Hesitancy and Fostering Vaccine Confidence. Health Commun..

[19] Razai M.S., Chaudhry U.A.R., Doerholt K., Bauld L., Majeed A. (2021). COVID-19 Vaccination Hesitancy. BMJ.

[20] Hussain B., Latif A., Timmons S., Nkhoma K., Nellums L.B. (2022). Overcoming COVID-19 Vaccine Hesitancy among Ethnic Minorities: A Systematic Review of UK Studies. Vaccine.

[21] ESOMAR/GBRN ESOMAR/GRBN Guideline for Online Sample Quality. https://grbn.org/wp-content/uploads/2016/12/Online_Sample_Quality_Guideline.pdf.

[22] GUS Społeczeństwo Informacyjne w Polsce w 2020 Roku. https://stat.gov.pl/obszary-tematyczne/nauka-i-technika-spoleczenstwo-informacyjne/spoleczenstwo-informacyjne/spoleczenstwo-informacyjne-w-polsce-w-2020-roku,1,14.html.

[23] Callegaro M., Lozar Manfreda K., Vehovar V. (2015). Web Survey Methodology.

[24] Wickham H. (2016). Ggplot2: Elegant Graphics for Data Analysis.

[25] Chaudhuri K., Chakrabarti A., Chandan J.S., Bandyopadhyay S. (2022). COVID-19 Vaccine Hesitancy in the UK: A Longitudinal Household Cross-Sectional Study. BMC Public Health.

[26] Robertson E., Reeve K.S., Niedzwiedz C.L., Moore J., Blake M., Green M., Katikireddi S.V., Benzeval M.J. (2021). Predictors of COVID-19 Vaccine Hesitancy in the UK Household Longitudinal Study. Brain Behav. Immun..

[27] Williams L., Flowers P., McLeod J., Young D., Rollins L. (2021). The CATALYST Project Team the CATALYST Project Team Social Patterning and Stability of Intention to Accept a COVID-19 Vaccine in Scotland: Will Those Most at Risk Accept a Vaccine?. Vaccines.

[28] Di Giuseppe G., Pelullo C.P., Della Polla G., Montemurro M.V., Napolitano F., Pavia M., Angelillo I.F. (2021). Surveying Willingness toward SARS-CoV-2 Vaccination of Healthcare Workers in Italy. Expert Rev. Vaccines.

[29] Wang J., Lu X., Lai X., Lyu Y., Zhang H., Fenghuang Y., Jing R., Li L., Yu W., Fang H. (2021). The Changing Acceptance of COVID-19 Vaccination in Different Epidemic Phases in China: A Longitudinal Study. Vaccines.

[30] Grochowska M., Ratajczak A., Zdunek G., Adamiec A., Waszkiewicz P., Feleszko W. (2021). A Comparison of the Level of Acceptance and Hesitancy towards the Influenza Vaccine and the Forthcoming COVID-19 Vaccine in the Medical Community. Vaccines.

