# Supplementary material for: Public Vaccination Reluctance: What Makes Us Change Our Minds? Results of A Longitudinal Cohort Survey
Source: Vaccines (Basel). 2022 Jul 5;10(7):1081. doi: 10.3390/vaccines10071081 (PMC9321479; doi:10.3390/vaccines10071081)
Supplement: Supplementary file 1 [file vaccines-10-01081-s001.zip › vaccines-1777310-supplementary.pdf]

SUPLEMENTARY MATERIAL

Original questions (in Polish)

( ) Pytanie jednokrotnego wyboru

P1. Czy jesteś zaszczepiony/-a przeciwko COVID-19?

Tak, jestem w pełni zaszczepiony/-a (dwiema dawkami lub jedną J& J)

Jestem po pierwszej dawce szczepionki i planuję przyjąć drugą

Jestem po pierwszej dawce szczepionki i nie planuję przyjąć drugiej

Nie jestem zaszczepiony/-a, ale planuję się zaszczepić (w ciągu najbliższych 6 miesięcy)

Nie jestem zaszczepiony/-a i nie planuję się zaszczepić (w ciągu najbliższych 6 miesięcy)

Zadaj jeśli P1=3 lub P1=5

[ ] Pytanie otwarte

P2. Dlaczego nie planujesz się zaszczepić przeciwko koronawirusowi?

[ ]

Zadaj jeśli P1=3 lub P1=5

[ ] Pytanie jednokrotnego wyboru + otwarte

P2_A. Czy jest coś, co mogłoby Cię skłonić do zmiany decyzji i zaszczepienia się?

Tak – co? Zapisz [ ]

Nie

Zadaj jeśli P1=1,2 lub 4

[ ] Pytanie otwarte

P3. Dlaczego zaszczepiłeś/-aś się / planujesz się zaszczepić przeciwko koronawirusowi?

[ ]

Translation into English

() Multiple choice question

P1. Are you vaccinated against COVID-19?

Yes, I am fully vaccinated (two doses or one J & J)

I am after the first dose of the vaccine and plan to take the second

I am after the first dose of the vaccine and have no plans to take the second

I am not vaccinated but plan to get vaccinated (in the next 6 months)

I am not vaccinated and I have no plans to vaccinate (in the next 6 months)

Ask if P1 = 3 or P1 = 5

[] Open-ended question

P2. Why are you not planning to get vaccinated against the coronavirus?

[]

Ask if P1 = 3 or P1 = 5 [] Multiple choice question + open-ended

P2_A. Is there anything that could make you change your mind and get vaccinated?

Yes - what? []

Not

Ask if P1 = 1,2 or 4

[] Open-ended question

P3. Why did you get vaccinated / plan to get vaccinated against coronavirus? []
